# Case report: A patient with mitochondrial neurogastrointestinal encephalomyopathy and chronic intestinal failure

**DOI:** 10.3389/fnut.2022.983873

**Published:** 2022-11-07

**Authors:** Ana Barisic, Dina Ljubas Kelecic, Darija Vranesic Bender, Irena Karas, Marko Brinar, Vladimir Miletic, Zeljko Krznaric

**Affiliations:** ^1^Clinical Unit of Clinical Nutrition, Department of Internal Medicine, University Hospital Centre Zagreb, Zagreb, Croatia; ^2^School of Medicine, University of Zagreb, Zagreb, Croatia; ^3^Division of Gastroenterology, Department of Internal Medicine, University Hospital Centre Zagreb, Zagreb, Croatia; ^4^Department of Neurology, University Hospital Centre Zagreb, Zagreb, Croatia

**Keywords:** mitochondrial neurogastrointestinal encephalomyopathy, malnutrition, home parenteral nutrition, intestinal failure, nutrition team

## Abstract

Mitochondrial neurogastrointestinal encephalomyopathy (MNGIE) is a rare disorder commonly diagnosed in later disease stages when it prominently manifests as malnutrition. We report on a female patient diagnosed with MNGIE at the age of 36. She was severely malnourished due to loss of resorptive surface after several surgical procedures, gastrointestinal dysmotility, and small intestinal bacterial overgrowth. Therefore, early and aggressive total parenteral nutrition was introduced. Although no reports have shown that nutritional support can modify the clinical outcome, this case suggests that adequate nutritional support, particularly parenteral nutrition, supervised by an experienced nutritional team, may prolong the lifespan of patients with MNGIE.

## Introduction

Mitochondrial neurogastrointestinal encephalomyopathy (MNGIE) is an extremely rare autosomal recessive multisystem disease, caused by pathogenic mutations in the *TYMP* gene. It is characterized by severe gastrointestinal dysmotility leading to cachexia, ptosis, external ophthalmoplegia, peripheral neuropathy, and leukoencephalopathy. A better understanding of the clinical heterogeneity of MNGIE is necessary in order to diagnose atypical cases, avoid excessive diagnostic procedures, and promote early diagnosis. MNGIE is diagnosed by testing for thymidine and deoxyuridine in the urine and plasma, as well as by Sanger sequencing of the *TYMP* gene. More than 60% of patients develop the disease before the age of 20, and the mean age at onset is 18 years. MNGIE has a poor prognosis, with the mean age of death of 35 years (range 15–54 years) (1). Patients with MNGIE usually die from severe malnutrition and gastrointestinal complications, therefore, nutritional interventions may be crucial in the management of these patients.

Herein, we present clinical, neuroimaging, and molecular findings of a patient with MNGIE who developed multifactorial chronic intestinal failure and required long-term home parenteral nutrition, which was shown to be invaluable in the management of this patient.

## Case report

A 36-year-old female patient with diabetes mellitus and diverticulosis of the small bowel was first referred to the Department of Gastroenterology and Hepatology at the University Hospital Center Zagreb in 2015. The patient had a history of multiple surgical procedures due to diverticulitis complications. She was transferred from a local hospital, where she had recently undergone a resection of a short ileal segment and creation of an ileoascending laterolateral anastomosis.

The patient reported more than 10 episodes of non-bloody, non-mucoid diarrhea, diffuse abdominal pain, vomiting, and bilateral hand tremor in the past month. She also reported having had bilateral ptosis and ophthalmoplegia in the past 2 years. Physical examination showed severe malnutrition, with body mass index (BMI) of 12.7 kg/m^2^. During the initial neurological examination, the patient was fully alert and oriented. Bilateral ptosis, limited horizontal and vertical eye movements, and postural “jerky” hand tremor were observed. The rest of the neurological examination was unremarkable.

Endoscopy and cross-sectional imaging of the gastrointestinal tract revealed stenotic ileocolonic anastomosis, diverticulosis of the small bowel, and suspected fistula in the blind loop of the colon.

**FIGURE 1:**
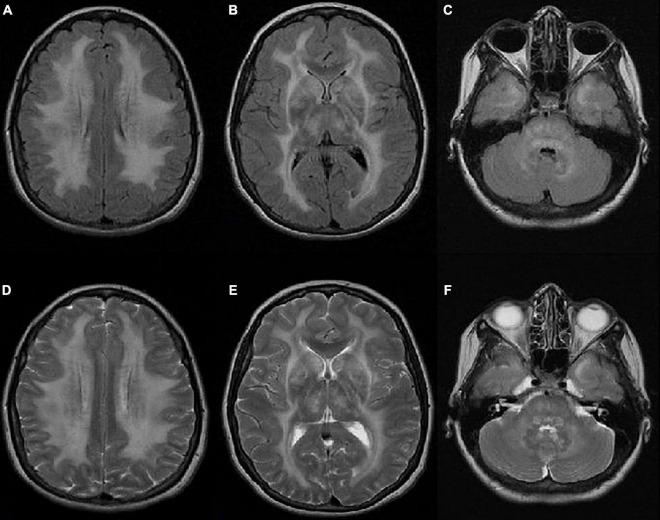
Axial FLAIR **(A–C)** and T2-weighted **(D–F)** MR images showing bilateral hyperintensities and edema in the basal ganglia. Diffuse leukoencephalopathy is visible with hyperintensities of the periventricular, subcortical, and deep white matter, including the pons and white matter of the cerebellum. There is no pathological imbibition of contrast or diffusion restriction.

Brain FLAIR and T2-weighted MR images demonstrated basal bilateral hyperintensities and edema in the basal ganglia. Diffuse leukoencephalopathy was visible, with hyperintensities of the periventricular, subcortical, and deep white matter, including the pons and white matter of the cerebellum. There was no pathological imbibition of contrast or diffusion restriction (Figure 1).

**FIGURE 2:**
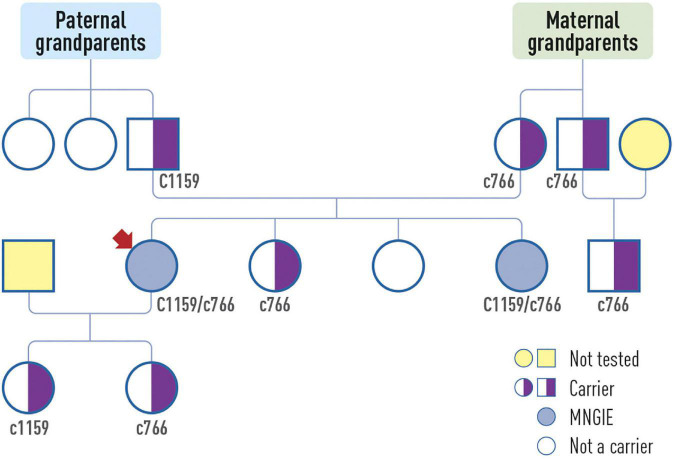
The patient’s (arrow) pedigree.

Electromyography revealed demyelinating sensory-motor polyneuropathy. Muscle biopsy showed no significant abnormalities. Genetic testing performed on suspicion of MNGIE identified two heterozygous pathogenic variants in the *TYMP* gene: c.1159 + 1G > A and c766G > T (p.Val256Phe). Genetic testing of the family members revealed MNGIE in one sibling, while the another sibling was a carrier (Figure 2). The affected sibling, who lived abroad, died due to complications of hematopoietic stem cell transplant at the age of 35.

Since the patient was severely malnourished and had persistent diarrhea, parenteral nutrition was initiated. All-in-one admixtures with adequate caloric composition were used, with the addition of daily recommended doses of vitamins and trace elements. This was the only way to ensure that the daily caloric and nutrient needs are met. Simultaneously, due to suspected small intestinal bacterial overgrowth (SIBO), antibiotic treatment was started.

As obstructive symptoms persisted, the patient underwent elective explorative laparotomy, which revealed severe diverticulosis of the small bowel, multiple adhesions, and stenosis of the anastomosis. Anastomosis resection was performed, followed by ileostomy and distal mucous fistula. In the late postoperative period, the patient developed gram-negative sepsis and an abscess in the abdominal wall. Another explorative laparotomy revealed perforated ileal loop; therefore, patch plasty and abscess drainage were performed. High-output stoma was treated with loperamide (16 mg), proton pump inhibitor (pantoprazole 2 × 40 mg), fluid resuscitation, correction of electrolyte abnormalities, octreotide (3 × 0.1 mL subcutaneous), parenteral nutrition (OlimelN9^®^ 1070 kcal/1000 mL, AA 1.53 g/kg/day, total energy 28.9 kcal/kg/day, lipid 1g/kg/day) in combination with enteral nutrition (Diasip^®^ 2 × 200 mL, total energy 400 kcal) and diet therapy. In addition to long-acting insulin preparations and rapid-acting insulin analog at meal times, 0.2 IU of rapid-acting insulin units per gram of dextrose was added directly to the parenteral nutrition.

Despite antisecretory therapy, stomal output remained high; therefore, the patient underwent stomal closure and ileotransverse anastomosis. Postoperatively, diarrhea persisted. The patient’s nutritional status remained poor, with no considerable improvement as multiple resections of the gut led to loss of intestinal absorptive capacity. Therefore, it was decided to start home parenteral nutrition and hydration. A port-A-catheter was placed, and the patient was discharged after 10 months of continuous hospital stay. During two years of home parenteral nutrition, two complications related to this nutrition regimen occurred (catheter-related blood stream infection and catheter dysfunction).

In June 2017, after we lost our patient from the follow-up for one year, she was admitted to our Neurology Department due to vision loss. Her nutritional status was impaired (BMI 11.9 kg/m^2^). Brain MRI showed the same changes as at initial presentation. Upon admission, total parenteral nutrition was started, and after three days the patient’s vision suddenly improved. Later, the patient stated that vitamins were not regularly added to her parenteral nutrition admixture. As her serum vitamin A levels were undetectable, peroral vitamin A supplements (900 mcg daily) were started, and regular IV vitamin intake in the parenteral nutrition admixture was continued.

In February 2020, the patient was admitted to our Unit due to sepsis, dysphagia, worsening of tremor, and muscle atrophy. Chronic diarrhea and abdominal pain accompanied with dysphagia further impaired her oral intake and worsened her nutritional status (with BMI 12 kg/m^2^). All this required a modification of home parenteral nutrition in terms of non-protein energy intake. However, due to quality-of-life and family issues, the patient was not inclined to IV feeding longer than 12–14 h per day, and a parenteral nutrition regimen was devised that was similar to the previous one (SMOF Kabiven^®^, 1100 kcal/986 mL, AA 1,47 g/kg/day, total energy 29.4 kcal/kg/day, lipid 1.1 g/kg/day) with additional dietary modifications. Urinary infection caused by extended-spectrum beta-lactamase-producing *Serratia marcescens* was treated with ertapenem, and prophylaxis with fosfomycin was administered. In addition, due to poorly controlled glucose levels, insulin therapy was corrected.

**FIGURE 3:**
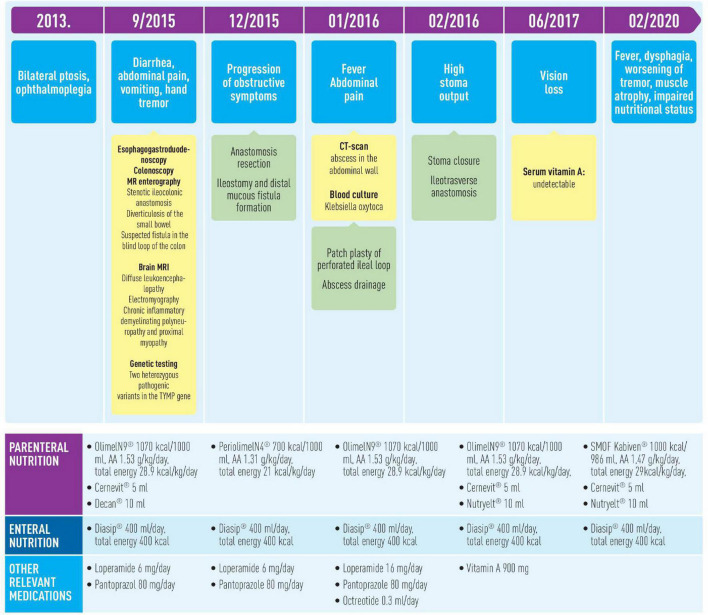
Timeline of interventions and outcomes.

Despite poor initial prognosis, the patient is currently 43 years old and has been on home parenteral nutrition for more than 6 years with acceptable quality of life (Figure 3). At the moment of writing, the patient’s nutritional (current BMI 12.9 kg/m^2^) and functional impairment continue to progress, but she is able to tolerate minimum oral intake and needs minimum assistance with functional mobility.

## Discussion

Mitochondrial neurogastrointestinal encephalomyopathy (MNGIE) is an extremely rare multisystem disease, with an estimated worldwide prevalence of <10 in a million. The number of patients with MNGIE is around 200 in the world (2, 3). A highly varying age at onset and various extent of involvement of several organ systems make it very difficult to establish the initial diagnosis, and the disease is usually misdiagnosed (4).

The major clinical features involve peripheral neuropathy, ocular symptoms, asymptomatic diffuse leucoencephalopathy, and gastrointestinal dysfunctions, such as gastrointestinal dysmotility, dysphagia, gastroesophageal reflux, nausea, postprandial emesis, abdominal pain and distension, and diarrhea. All these features cause progressive weight loss and severe malnutrition (5). MNGIE patients have been sporadically reported to present with endocrine dysfunction, such as diabetes, as is the case with our patient (1). These patients may also develop complications, including diverticular ruptures, intestinal perforations, and aspiration pneumonia, that expose them to infections leading to a fatal outcome (1).

The gold standard of MNGIE diagnostics is testing for thymidine and deoxyuridine in the urine and plasma, as well as Sanger sequencing of the *TYMP* gene. However, the complex clinical picture often leads to diagnostic delays and unnecessary invasive diagnostic procedures. When novel variants of the *TYMP* gene are identified or Sanger sequencing of *TYMP* is unavailable, thymidine phosphorylase activity needs to be evaluated. This evaluation may also serve as a complement to the measurement of thymidine and deoxyuridine concentrations in body fluids. Targeted gene testing for primary *TYMP* mutations or more comprehensive genomic analyses for the whole genome including secondary mtDNA mutations can be used, such as Sanger or next generation sequencing, Southern blot, quantitative PCR, multiplex ligation-dependent probe amplification, and genome-wide single nucleotide polymorphism microarrays (6).

Currently, there are no specific therapies for MNGIE. Disease management is mainly symptomatic and requires multidisciplinary approach. Gastrointestinal symptoms are treated with analgesics, anti-emetics, prokinetics, and antibiotics for intestinal bacterial overgrowth. Limb pain caused by peripheral polyneuropathy is treated with centrally acting agents, such as amitriptyline, gabapentin, and pregabalin (7).

Several experimental therapeutic approaches are currently under investigation. Most of them, by reducing or eliminating thymidine and deoxyuridine, ameliorate intracellular deoxyribonucleoside imbalances and prevent further damage to mtDNA.

Biochemical imbalance can be temporarily improved by hemodialysis, continuous ambulatory peritoneal dialysis, erythrocyte encapsulated thymidine phosphorylase infusion, and platelet infusion (8). On the other hand, allogeneic hematopoietic stem cell transplantation (9) and orthotopic liver transplant (10) have been shown to permanently restore the biochemical imbalance (8). However, these treatments carry a high risk of complications and mortality, especially in severely malnourished patients (8). Bearing this in mind, as well as bearing in mind the fact that the patient was diagnosed in an advanced stage of the disease, our multidisciplinary team concluded that none of these treatments was suitable.

Because a majority of MNGIE patients die due to complications of malnutrition, which often develops in later stages of the disease, adequate nutritional support is of paramount importance. Severe malnutrition in our patient was multifactorial, mainly caused by short bowel (loss of resorptive surface of the ileum and the right colon, without the presence of the ileocecal valve), GI dysmotility, and possibly SIBO (which is commonly present in patients with GI dysmotility). Although no reports showed that nutritional support can modify the clinical outcome (11–13), parenteral nutrition was lifesaving for our patient with chronic intestinal failure. Optimal nutritional support can also help to improve poor nutritional status, thus maximizing the benefits of experimental therapeutic approaches and reducing the risk of complications. Long-term parenteral nutrition together with chronic intestinal failure may lead to a number of challenges and complications, the most important being intestinal failure-associated liver disease and sepsis. It is a controversial issue whether MNGIE patients are at risk of metabolic oversupply from the lipid and carbohydrate components of parenteral nutrition, which can cause further mitochondrial toxicity (5). Therefore, we would like to stress the importance of a continuous involvement of an experienced nutritional support team in the management of these patients. Our patient was receiving 1.1 g/kg/day of AA and 3.68 g/kg/day of dextrose, and her liver function tests remained normal while she was on home parenteral nutrition. The patient was lost to follow-up for one year. During this time, she did not receive adequate vitamin and mineral supplementation, which resulted in severe vitamin A deficiency and vision loss.

## Conclusion

This case highlights the importance of adequate nutritional support and monitoring in patients with MNGIE, in particular of parenteral nutrition in later stages of the disease. An optimized nutritional status might translate into improved survival of patients with this complex disease.

## Data availability statement

The original contributions presented in this study are included in the article/supplementary material, further inquiries can be directed to the corresponding author.

## Ethics statement

The patient provided written informed consent for study participation and the publication of any potentially identifiable images or data included in this article.

## Author contributions

AB and DL wrote the manuscript. All authors participated in the diagnosis of disease, medical treatment, follow-up of the patient, read, and approved the final manuscript.
